# Identification and Characterization of Five Cold Stress-Related Rhododendron Dehydrin Genes: Spotlight on a FSK-Type Dehydrin With Multiple F-Segments

**DOI:** 10.3389/fbioe.2019.00030

**Published:** 2019-02-21

**Authors:** Hui Wei, Yongfu Yang, Michael E. Himmel, Melvin P. Tucker, Shi-You Ding, Shihui Yang, Rajeev Arora

**Affiliations:** ^1^National Renewable Energy Laboratory, Biosciences Center, Golden, CO, United States; ^2^Department of Horticulture, Iowa State University, Ames, IA, United States; ^3^State Key Laboratory of Biocatalysis and Enzyme Engineering, Hubei Collaborative Innovation Center for Green Transformation of Bio-Resources, Environmental Microbial Technology Center of Hubei Province, Hubei Key Laboratory of Industrial Biotechnology, School of Life Sciences, Hubei University, Wuhan, China; ^4^National Renewable Energy Laboratory, National Bioenergy Center, Golden, CO, United States; ^5^DOE-Great Lakes Bioenergy Research Center, Michigan State University, East Lansing, MI, United States; ^6^Department of Plant Biology, Michigan State University, East Lansing, MI, United States

**Keywords:** expressed sequence tags (EST), gene expression profiling, cold hardiness, *Rhododendron*, cold acclimation, deacclimation, FSK-type dehydrins, dehydrin F-segment

## Abstract

Dehydrins are a family of plant proteins that accumulate in response to dehydration stresses, such as low temperature, drought, high salinity, or during seed maturation. We have previously constructed cDNA libraries from *Rhododendron catawbiense* leaves of naturally non-acclimated (NA; leaf LT_50_, temperature that results in 50% injury of maximum, approximately −7°C) and cold-acclimated (CA; leaf LT_50_ approximately −50°C) plants and analyzed expressed sequence tags (ESTs). Five ESTs were identified as dehydrin genes. Their full-length cDNA sequences were obtained and designated as *RcDhn 1-5*. To explore their functionality vis-à-vis winter hardiness, their seasonal expression kinetics was studied at two levels. Firstly, in leaves of *R. catawbiense* collected from the NA, CA, and de-acclimated (DA) plants corresponding to summer, winter and spring, respectively. Secondly, in leaves collected monthly from August through February, which progressively increased freezing tolerance from summer through mid-winter. The expression pattern data indicated that *RcDhn 1-5* had 6- to 15-fold up-regulation during the cold acclimation process, followed by substantial down-regulation during deacclimation (even back to NA levels for some). Interestingly, our data shows RcDhn 5 contains a histidine-rich motif near N-terminus, a characteristic of metal-binding dehydrins. Equally important, RcDhn 2 contains a consensus 18 amino acid sequence (i.e., ETKDRGLFDFLGKKEEEE) near the N-terminus, with two additional copies upstream, and it is the most acidic (pI of 4.8) among the five RcDhns found. The core of this consensus 18 amino acid sequence is a 11-residue amino acid sequence (DRGLFDFLGKK), recently designated in the literature as the F-segment (based on the pair of hydrophobic F residues it contains). Furthermore, the 208 orthologs of F-segment-containing RcDhn 2 were identified across a broad range of species in GenBank database. This study expands our knowledge about the types of F-segment from the literature-reported single F-segment dehydrins (FSK_n_) to two or three F-segment dehydrins: *Camelina sativa* dehydrin ERD14 as F_2_S_2_K_n_ type; and RcDhn 2 as F_3_SK_n_ type identified here. Our results also indicate some consensus amino acid sequences flanking the core F-segment in dehydrins. Implications for these cold-responsive RcDhn genes in future genetic engineering efforts to improve plant cold hardiness are discussed.

## Introduction

Survival and growth of woody plants in cold climate is important for traditional sectors of horticulture and forestry. One advantage of using Rhododendron as a material to study cold-hardiness physiology is the wide range of leaf and bud cold (freezing) tolerance among species (Sakai, 1986). *Rhododendron*, like many other woody perennials, can adapt to harsh winter through a process called cold acclimation (CA), by which they develop tolerance to low temperature and freezing seasonally, with hardiness increasing through the autumn, peaking in midwinter, declining during the spring, and reaching the lowest in summer (Arora and Taulavuori, 2016).

Cold acclimation is considered to be an active process that involves a wide range of physiological and biochemical reprogramming, including altered membrane structure and function (Yamada et al., 2002), as well as myriad of changes in primary and secondary metabolisms (Guy, 1990; Thomashow, 1990, 1998); most of these are also accompanied by related changes in protein/gene expression. As the D-11 subgroup of late embryogenesis abundant proteins (Dure, 1993), dehydrins have been found to play an important role in plant defense against dehydration stresses, including freeze-desiccation stress (Lin and Thomashow, 1992; Close, 1996; Wisniewski et al., 2003; Kaplan et al., 2004; Kosová et al., 2007; Tunnacliffe and Wise, 2007). The defining characteristic of plant dehydrins is the existence of a putative amphipathic α-helix-forming domain, called the conserved K-segment (Close, 1997; Malik et al., 2017). It has been shown that dehydrins are located in the nucleus or cytoplasm of the cell (Close, 1997), specifically in the vicinity of the plasma membrane (Danyluk et al., 1998), cytoplasmic endomembrane (Egerton-Warburton et al., 1997), and plasmodesmata (Karlson et al., 2003). In addition, their high concentrations in cells (Baker et al., 1988), add to the appeal as engineering targets for enhancing plant stress defense capacity.

We had previously generated 862 5′-end high-quality ESTs from cold acclimated (CA) and non-acclimated (NA; non-cold-hardened) leaves of field grown plants of *Rhododendron catawbiense*, a cold-hardy North American rhododendron species (Wei et al., 2005a). NA (summer-collected) and CA (winter-collected) leaves were also evaluated for cold-hardiness in a laboratory-based freeze-thaw assay which indicated their leaf-freezing tolerance (defined as LT_50_, temperature that results in 50% injury of maximum) to be approximately −7° and −50°C, respectively. Comparative analysis of NA- and CA-EST data sets revealed cDNAs for five dehydrins that were more abundant in the more cold-hardy CA tissues (Wei et al., 2005a), and are thus of interest for further characterization. In the present study, sequence analyses of these five rhododendron dehydrins were performed to characterize their conserved motif features. In addition, the seasonal gene expression of individual dehydrins was characterized using northern blot and RT-PCR, providing experimental information on their cold-acclimation-response. Furthermore, a thorough bioinformatic analysis was carried out for an identified 18 amino acid sequence (ETKDRGLFDFLGKKEEEE) located in one of the rhododendron dehydrins. Interestingly, the center part of this consensus 18 amino acid sequence is a 11-residue amino acid sequence (DRGLFDFLGKK) that has been recently identified and named as the “F-segment” based on the pair of hydrophobic F residues it contains (Strimbeck, 2017). This present study, however, expands our knowledge regarding the types of F-segment peptides found in the known single copy (FSK_n_) dehydrins to the F_2_S_2_K_n_ or F_3_SK_n_ dehydrins which contain two or three F-segments (this study). Our bioinformatic analysis also indicates some consensus amino acid sequences flanking the core F-segment in at least some of the F-segment containing dehydrins. Potential use of the identified cold stress-related rhododendron dehydrins for plant engineering is also discussed.

## Materials and Methods

**Figure 1:**
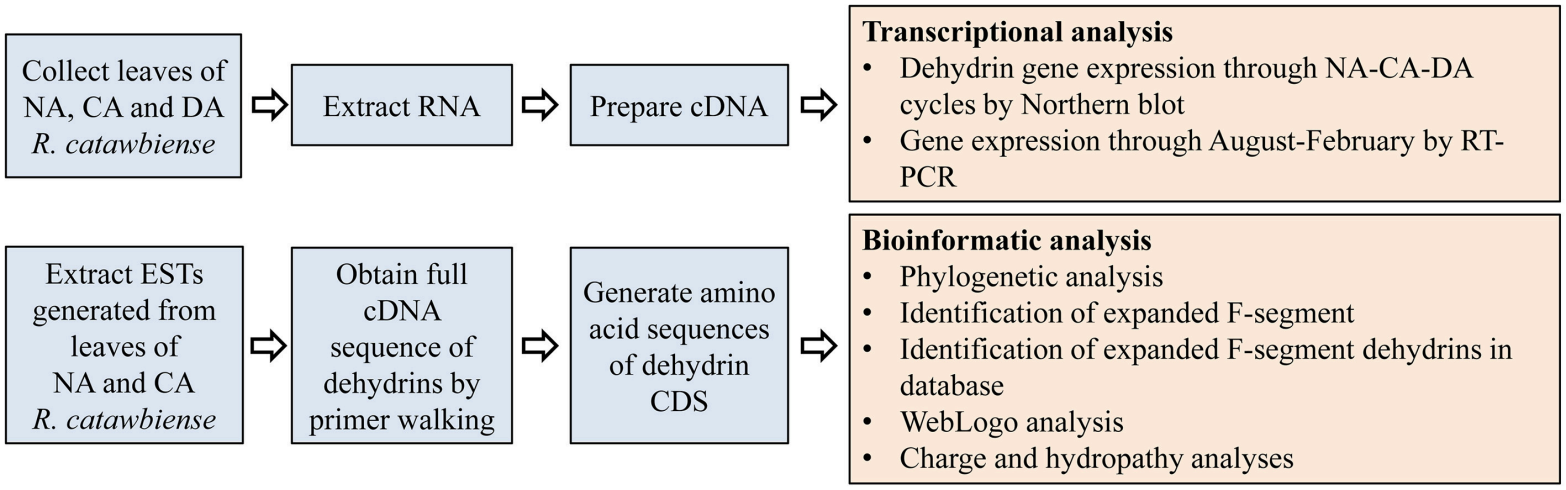
Diagram of the outline for the experimental design and analyses.: CA, cold-acclimated; DA, de-acclimated; EST, expressed sequence tag; NA, non-acclimated.

The overall experimental and analysis approaches are illustrated in Figure 1.

### Sample Collection

Field-grown plants of *R. catawbiense*, maintained at The Holden Arboretum's David G. Leach Research Station in Madison, Ohio, were used for this study. Two sets of leaf samples were collected from these plants to determine the changes of dehydrin expression profiles. The first set of leaf samples was the seasonal collection representing summer (July), winter (January), and the following spring (May). Summer and winter-collected samples represent NA and CA leaves, respectively; whereas the spring collection is for tissues that are expected to have lost their previously acquired (during fall/winter) cold hardiness in a process called deacclimation (DA) upon the return of warmer temperatures in spring (Kalberer et al., 2006). Together, this sampling represented annual cycle of NA-CA-DA tissues. Leaf freezing tolerance, defined as LT_50_, of NA and CA leaves, was found to be −7° and −53°C, respectively (Wei et al., 2005a). Whereas precise freezing tolerance of deacclimated leaves (May collection) could not be ascertained for this study, it can be safely assumed to be substantially lower than cold acclimated levels (from January) and closer to that of non-acclimated tissues (Kalberer et al., 2006). The second set of leaf samples was approximately monthly collections from August through February, representing the period of gradual/seasonal development of cold acclimation from summer (August) through the fall/early winter (September, October, November) reaching close to maximal cold-hardiness by January. For all the samplings, leaf tissues were flash frozen in liquid nitrogen and stored at −80°C until RNA and cDNA preparations.

### RNA Extraction

Total RNA was extracted according to the modified hot-borate method of Wilkins and Smart (Wilkins and Smart, 1996). The prepared RNA was dissolved in DEPC treated water and store at −80°C until use.

### Northern Blot

Equal amounts of total RNA (8 μg) extracted from leaf tissues were denatured and fractionated on 1% (w/v) formaldehyde-agarose gels for electrophoresis, followed by viewing and photographing under UV light to confirm RNA quality and equal sample loading. The transfer of RNA to nylon membranes, the preparation of DNA probes corresponding to cDNA inserts of interest, and the hybridization conditions were described previously (Wei et al., 2005a). After the northern blotting, the intensity of positive bands was analyzed by densitometry using imaging software (NIH Image version 1.41, National Institutes of Health, Bethesda, MD).

### Reverse Transcription

For each sample's reverse transcription (RT), RNA was treated with DNase I (amplification grade; Invitrogen, Carlsbad, CA) to avoid contamination with genomic DNA. First-strand cDNA was synthesized using 3 μg of total RNA with the Superscript RT III kit (Invitrogen, Carlsbad, CA) and random hexamer primers for 18S and *R. catawbiense* ubiquitin-like (*RcUbql*) genes (used for initial reference gene screening), or oligo(dT)18 for *RcUbql* gene and dehydrin genes (used in formal functional gene screening) according to the manufacturer's instructions. The total RT reaction volume was 20 μL and was further diluted to 80 μL by adding DEPC-treated water (thus each μL contained “first-strand cDNA” derived from approximately 40 ng of initial total RNA). This was used as “first-strand cDNA” for regular RT-PCR and real-time RT-PCR as described below. A second aliquot of total RNA (also 3 μg) was treated using ddH_2_O instead of reverse transcriptase and used as minus reverse transcriptase (–RT) controls for monitoring any genomic DNA contamination or nonspecific DNA amplification.

### Selection and Validation of *RcUbql* as Reference Genes for Regular and Real-Time RT-PCR

As in our EST dataset, we have identified the EST for *RcUbql* (GenBank accession No. CV015651), which allowed us to design a pair of primers for both regular RT-PCR and real-time RT-PCR with an amplicon size of 254 bp (Table 1). To validate the suitability of *RcUbql* as the reference gene, the Quantum RNA Universal 18S Internal Standards primers (amplicon size of 315 bp; Ambion, Austin, TX, USA) were used as an internal standard with 18S primers-to-competimers ratio of 3:7. As described by the manufacturer's manual, the 18S rRNA and our target gene (*RcUbql*) were amplified in a multiplex reaction using the above-mentioned random hexamer primers-reverse transcribed cDNA as templates.

**Table 1:** Primer sequences for regular and real-time RT-PCR.

***Dhn* genes**	**Forward primer**	**Amplicon size (bp)**
*RcDhn* 1	F: CCACCAGTCCCACGACACTA R: TACCCACCACCTGCTCCAG	57
*RcDhn* 2	F: AAGGATGGGTTGTTGACGAAGT R: TTCCTCCGAAGAGCTTGAGC	51
*RcDhn* 3	F: ATCGCCCCGTCCTAATCTTCT R: CCCTCGAGACTCCGTCCAC	71
*RcDhn* 4	F: CGTGGACAAGGTGAAGGACAA R: ACTAGCGGCGGAAAAGAAGAT	111
*RcDhn* 5	F: AAGTTCCACCGTTCCGATAGC R: ATTCGTGTCCTCCTCGTGCT	128
*RcUbql*	F: AGAGGTGGTGTTGAACGATCG R: TCTCGCACTTATTACCGCACA	254

*GenBank accession No. for RcDhn 1-5 can be found in Table 2, while that for RcUbql is CV015651*.

### Regular RT-PCR and Real-Time RT-PCR

The primers used for both regular and real-time RT-PCR are listed in Table 1. Whereas, the regular RT-PCR with three different cycle numbers provided a visual, traditional means to examine the expression level of target genes, the real-time RT-PCR allowed a more accurate, quantitative assessment of the gene expressions.

For regular RT-PCR, which was used in parallel to real-time RT-PCR (as described below) to detect the expression level of dehydrin genes and *RcUbql* gene, the “first-strand cDNA” (derived from approximately 40 ng of initial total RNA) was used in a final reaction of 20 μL containing 0.2 mM dNTP, 2 mM MgCl_2_, 625 nM of each forward and reverse primers and 1 unit of Taq. The setup reaction mixture was subjected to regular RT-PCR at three different cycle numbers empirically determined for amplification at non-saturation levels (28, 32, and 36 cycles for most transcripts). This setup ensured that the amount of amplified products stayed in the linear proportion to the initial template amount present in the reaction under at least one of these three cycle numbers. The PCR products were separated and analyzed on agarose gels.

For real-time RT-PCR, the “first-strand cDNA” (equivalent to approximately 10 ng of initial total RNA extracted from leaf tissues) was used in a final reaction of 20 μL containing 1X SYBR Master Mix, 625 nM forward primer and 625 nM reverse primer, using ABI optical tube and caps. All reactions were performed in triplicate and repeated in two independent experiments. The real-time RT-PCR were performed in ABI model 7000 sequence detection system (Applied Biosystems, Foster City, CA). Thermal cycling conditions consisted of 2 min at 94°C for denaturation and 40 cycles of amplification (15 s at 94°C, 30 s at 59°C, 20 s at 72°C), followed by standard dissociation procedure. PCR data were analyzed with the sequence detection software version 1.2.3.

PCR amplification efficiency of real-time RT-PCR was determined using the absolute fluorescence method (Ramakers et al., 2003), in which a serial cDNA template dilutions were conducted to obtain the standard curves. The resultant PCR efficiency for each gene's primers was calculated. Expression level of test gene (i.e., *RcDhn 1-4*) relative to reference gene (*RcUbql*) was calculated using the comparative CT method, i.e., by subtracting the CT of reference gene from the test gene CT according to the function ΔCT = CT (test gene)—CT (reference gene). To obtain the seasonal changes in expression levels of a certain *RcDhn* gene, the function ΔΔCT was determined using the equation ΔΔCT = ΔCT(test gene in a specific month's sample)—ΔCT(test gene in August sample). The final fold change of a specific month against August was then calculated by the formula 2^−ΔΔ*Ct*^ in accordance with ABI sequence detection system user manual, with the gene expression level in August set as 1. For statistical analysis, the *p*-values were calculated using a Student's *t*-test on the fold change values, and the analyses were performed using Excel; significance was defined as *p* < 0.05, whereas high significance was defined as *p* < 0.01.

### ESTs Source and Primer Walk to Obtain Full cDNA Sequence of Dehydrins

Previously, 423 and 439 5′ ESTs were generated from cold acclimated (CA) and non-acclimated (NA) leaves, respectively, of *R. catawbiense* (Wei et al., 2005a). These ESTs (GenBank accession nos. CV014938– CV015799) were clustered to produce a list of unique transcripts, which were annotated using PIR-NREF protein database (Protein Information Resource: Non-Redundant Reference) and BLASTX (Wei et al., 2005a). The study annotation led to the identification of five dehydrins, which were labeled as *RcDhn* 1-5, wherein “*Rc*” represents *R. catawbiense, Dhn* for dehydrin, and each gene has a unique number.

Sequences of full length dehydrin genes were obtained by primer-walking sequencing of the 5 *RcDhn* cDNA clones. The primers used were either the universal primers or designed based on the sequences of ESTs that also existed for cDNA clones. DNA multiple sequence alignments were conducted by using the Genetics Computer Group (GCG) PILEUP program (University of Wisconsin, Madison, WI, USA) to determine the full-length sequences of the cDNA clones.

### Open Reading Frame (ORF) of the Nucleotide Sequences and Protein Sequence Alignment

The full-length sequences of the *RcDhn* clones were input into the NCBI's ORF to deduce the amino acid sequences of the dehydrin genes. The most feasible ORF was determined by comparing the deduced amino acid sequence with the sequences in GenBank databases using the BLAST server. The resultant amino acid sequences were used to (1) identify the potential YSK segments of dehydrins, (2) identify the expanded F-segment (see below), and (3) align specific dehydrins of interest from other plant sources.

### Identification and Clustering of the Expanded F-Segment Containing Ortholog Proteins and Bioinformatics Analysis

**Figure 2:**
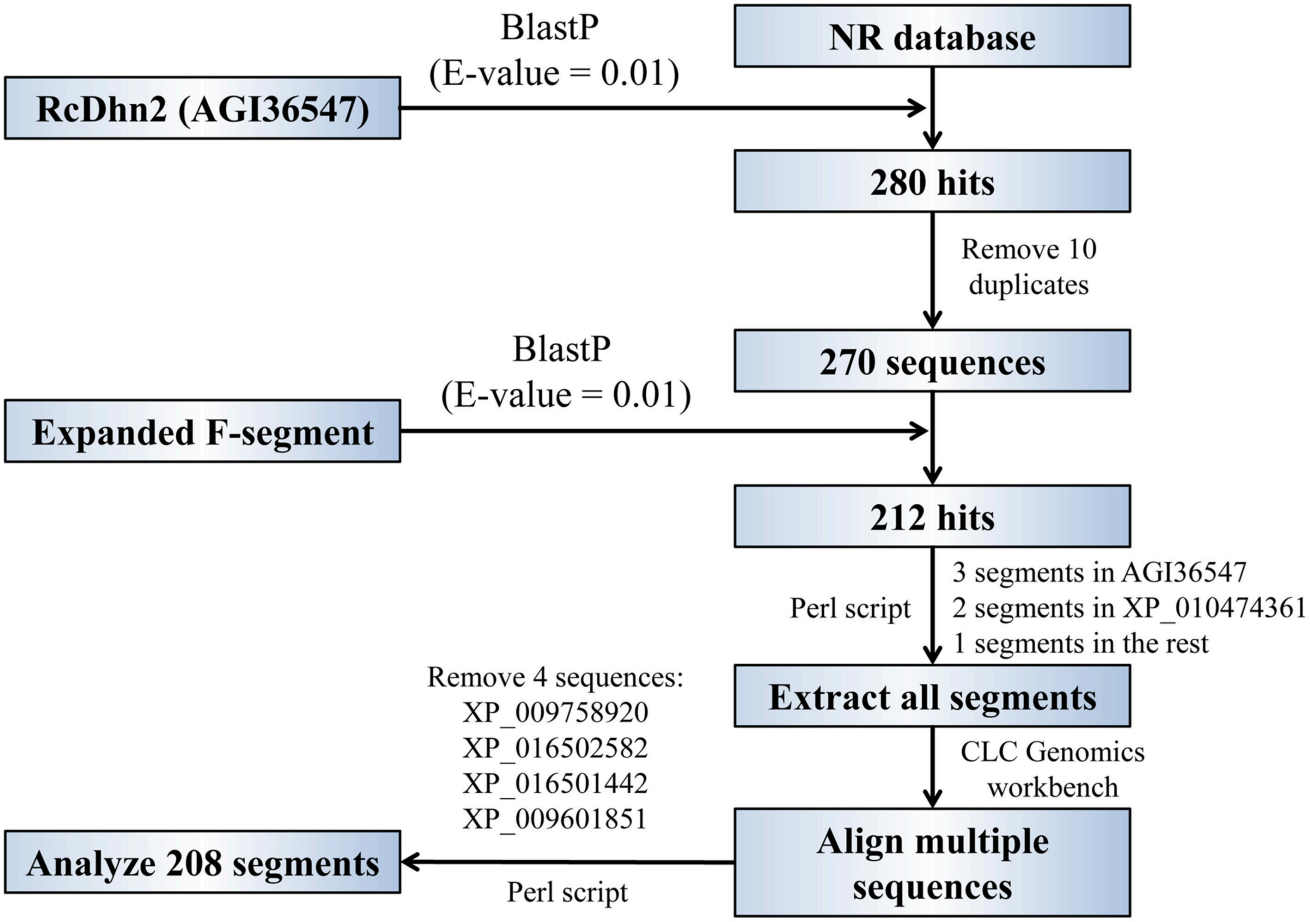
Flowchart for global and local BlastP analyses to identify the homologous proteins that contain the conserved, expanded F-segment in protein database. See the Materials and Methods section for details.

**Figure 3:**
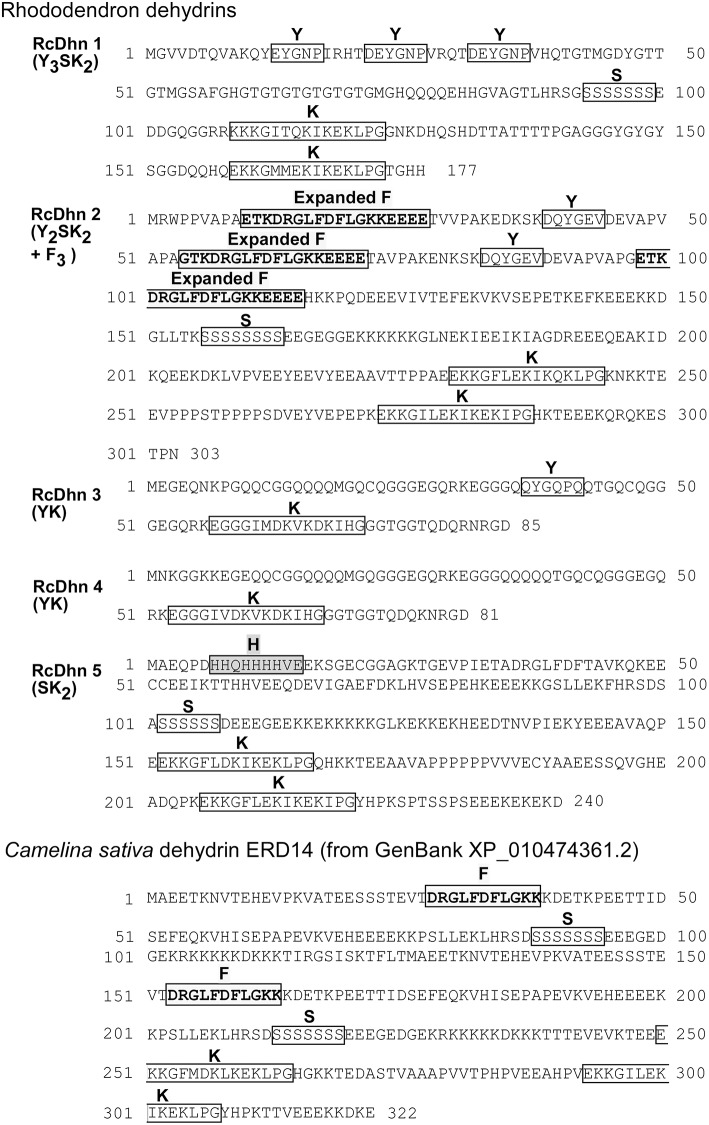
Deduced amino acid sequences for *Rhododendron* dehydrins RcDhn 1-5 and *C. sativa* dehydrin ERD14. Amino-acid residues are designated in single-letter code. The Y-, S-, and K-segments are boxed. The defined expanded F-segments in RcDhn 2 are boxed and in boldface and yellow; the histidine-rich (H) segment are boxed and in pink. *C. sativa* dehydrin ERD14 was identified by our BlastP analysis hit against the NCBI protein database.

The procedure for BlastP analyses to identify the homolog proteins that contain the conserved F-segment in protein database is outlined in Figure 2. The *R. catawbiense* dehydrin 2 (RcDhn 2) amino acid sequence obtained from NCBI (AGI36547) was used to search for other similar dehydrins using the local Protein-protein BLAST (BlastP) program against the non-redundant (NR) protein database. The e-value was set to 0.01 and other parameters kept at default. Consequently, 270 sequences were retained to do the next analysis after removing the 10 repeats in all 280 hits. Another local BlastP was performed to search for the similar amino acid consensus sequence using the 18 amino acid sequence initially identified in RcDhn 2 (ETKDRGLFDFLGKKEEEE) as the query and the 270 sequences as database. The e-value was set to 0.01 and other parameters kept at default. There were 212 hits with AGI36547 (i.e., RcDhn 2 deposited into GenBank by our group) containing three F-segments, XP_010474361 containing two F-segments (*Camelina sativa* dehydrin ERD14; F_2_S_2_K_n_ as illustrated in Figure 3, bottom panel), and the rest containing one F-segment.

The start and end positions of the 212 F-segments in the BlastP result was extracted using a customized Perl script, which were then used for multiple sequence alignment. Four segments containing two amino acid “CG” insertions may belong to another family and were removed for alignment, as illustrated in Supplemental Figure S1. The rest 208 segments were used for consensus sequence analysis and the unrooted evolutionary tree construction by using CLC Genomics workbench 9. The consensus sequence Logo was then constructed by WebLogo online server (http://weblogo.berkeley.edu/logo.cgi) (Schneider and Stephens, 1990; Crooks et al., 2004).

### Charge and Hydropathy Analyses of Conserved Segments of Dehydrins

For conserved segments, Peptide Analyzer (http://haubergs.com/peptide) was used to calculate the charge and hydropathy scores, and to generate hydropathy plot.

## Results

### Sequence Analyses of *Rhododendron* Dehydrin Genes

BLASTX search of PIR-NREF protein database revealed that several ESTs from the CA library were identified as dehydrin transcripts encoding five distinct dehydrins (Table 2). Five corresponding cDNA clones from the cold-acclimated cDNA library (Wei et al., 2005a) were picked and cultured for plasmid extraction. The extracted plasmid DNA was sent to the DNA facility of Iowa State University for Primer Walking service to obtain the full-length sequence of these clones. The resultant sequence analysis showed that each cDNA contains the 3′ untranslated region, the start codon, stop codon, and the poly(A)+ tail, confirming that they represent the full-length genes (data not shown). The deduced amino acid sequences from these genes are shown in Figure 3.

**Table 2:** Characteristics of *Rhododendron Dhn* genes and their protein products.

***Dhn* genes**	**GenBank Protein No. (cDNA clone No.)**	**Dehydrin type**	**Amino acid number**	**MW**	**PI (kDa)**	**Picked times^a^**
*RcDhn 1*	KC425881 (CA3A12)	Y_3_SK_2_ potato type	177	20	6.5	1
*RcDhn 2*	KC417479 (CA5B04)	Y_2_SK_2_ + F_3_	303	34	4.8	1
*RcDhn 3*	KC425882 (CA1F12)	Y_1_K_1_	85	10	6.9	4
*RcDhn 4*	KC425883 (CA3E05)	K_1_ blueberry type	81	10	6.9	1
*RcDhn 5*	ACB41781 (CA2D12)	SK_2_ kidney bean type	240	29	5.2	1

a *Number of times that a particular cDNA was picked from cDNA library (containing 423 5′ end ESTs) of rhododendron cold acclimated (CA) leaf tissues (Wei et al., 2005a)*.

As noted before, the distinct Dhn genes were referred by a nomenclature composed of five-letter (*RcDhn*) plus a sequential number (1 to 5), in which “*Rc*” represents *R. catawbiense, Dhn* for dehydrin. The dehydrin protein names follow the same convention, except that the letters are capitalized and not italicized. Based on the presence of certain consensus regions of amino acids in their sequence, dehydrins are conventionally described by the “YSK” shorthand, according to which plant dehydrins can be categorized into five distinct structural types: (1) Y_n_SK_n_, (2) SK_n_, (3) Y_n_K_n_, (4) K_n_, and (5) K_n_S (Close, 1996, 1997). Except for the type 5 (K_n_S), other four types, 1 to 4, have been identified in *Rhododendron* in this study (Table 2). The predicted size of dehydrins identified was from 81 to 303 in amino acids (Table 2), which fits within the reported wide range of dehydrins (82-648 amino acids) (Close, 1996). Variation in isoelectric point is in the range of 4.8 to 6.9. It is suggested that each YSK structure type may bear a distinctive functional role (Svensson et al., 2002), thus we characterized each identified dehydrin as below.

**(1) Y_3_SK_2_ type RcDhn 1**
  Y_3_SK_2_ has been found widely across diverse plant species some of which include sunflower (*Helianthus annuus*), radish (*Raphanus sativus*) (Campbell and Close, 1997), *Arabidopsis thaliana* (Nylander et al., 2001; Svensson et al., 2002), *Brassica juncea* and *B. napus* (Yao et al., 2005). The transcript of sunflower Y_3_SK_2_ type dehydrin (*HaDhn1*) increased in abundance under water deficit stress (Cellier et al., 2000), while this type of dehydrins in *B. juncea* and *B. napus* were found to be expressed in germinating seeds and with enhanced cold tolerance during seedling emergence (Yao et al., 2005).
**(2) Y_2_SK_2_ + F_3_ type RcDhn 2 (F_3_SK_2_)**
  RcDhn 2 (Z05B04) belongs to the Y_2_SK_2_ type and can be distinguished from the other four rhododendron dehydrins because it has three copies of an unusual, expanded F-segment (ETKDRGLFDFLGKKEEEE), one of which is always present near the N-terminus (Figure 3; Table 2). Dehydrins with all three consensus segments, Y, S, and K, have been widely reported to occur in plants (Close, 1996; Wisniewski et al., 2006).
**(3) RcDhn 3 and RcDhn 4: the blueberry-type dehydrins**
  RcDhn 3 (CA1F12; Y_1_K_1_) belongs to Y_1_K_1_ type (Figure 3 and Table 2). Although Y_n_K_n_ dehydrins have been found in other species, such as Y_2_K_2_ (*Pisum sativum*) (Haider, 2012), and Y_2_K_9_ (*Prunus persica*) (Wisniewski et al., 2006), Y_1_K_1_ type has not been reported in literature based on our knowledge. RcDhn 4 (CA3E05; K_1_) belongs to K_1_ type (Figure 3 and Table 2); unlike RcDhn 3, it lacks Y-segment. K_n_ type dehydrins have been found in many other species, including K_2_ in *Pseudotsuga menziesii*, K_3_ in *Medicago falcata*, K_6_ in *Triticum aestivum* and *A. thaliana*, and K_9_ in *Hordeum vulgare* (Campbell and Close, 1997). In addition, K_2_ type dehydrins also exist in *Pinus sylvestris* (GenBank accession No. CAD54624.1, CAD54623.1, and CAD54621.1). However, K_1_ type has not been reported in other species so far. Since the defining feature of dehydrins is the conserved K-segment, the K_1_ type RcDhn 4 is among the groups of a few simplest dehydrins reported so far with regard to deduced amino acid sequences(Lee et al., 2005). Except for the Y-segment, both RcDhns 3 and 4 have K_1_ and also show considerable similarity to each other in their deduced amino acid sequences. BLAST search against NR-PIR database identified five blueberry (*Vaccinium corymbosum*) dehydrin orthologs (Dhanaraj et al., 2005). Since *Rhododendron* and blueberries both belong to the health family (*Ericaceae*), RcDhns 3 and 4 were thus labeled as blueberry-type dehydrins.
**(4) RcDhn 5: the kidney bean-type dehydrins**
  RcDhn 5 (CA2D12; SK_2_) is an acidic, SK_2_ type dehydrin (Figure 3 and Table 2); it lacks the Y-segment. It also contains a histidine-rich segment (HHQHHHHVE) close to N-terminus. SK_2_ dehydrins have also been found in other woody plants like peach (*Prunus persica*) (GenBank accession No. AAZ83586) (Bassett et al., 2009) and birch in which the pre-exposure to short-day followed by low-temperature treatment led to a significant increase in the expression of a SK2 type dehydrin gene, compared with low-temperature-treated plants grown at long-day photoperiod (Puhakainen et al., 2004). Heterologous expression of this birch SK2 type dehydrin in *Arabidopsis* indicated that this short-day potentiation of gene expression could be tree-specific (Puhakainen et al., 2004).

### Seasonal Expression Profiling of RcDhn Genes by Northern Blot: Approach I to Identify Quantitative Expression of *RcDhns* vis-à-vis Seasonal Changes in Freezing Tolerance

**Figure 4:**
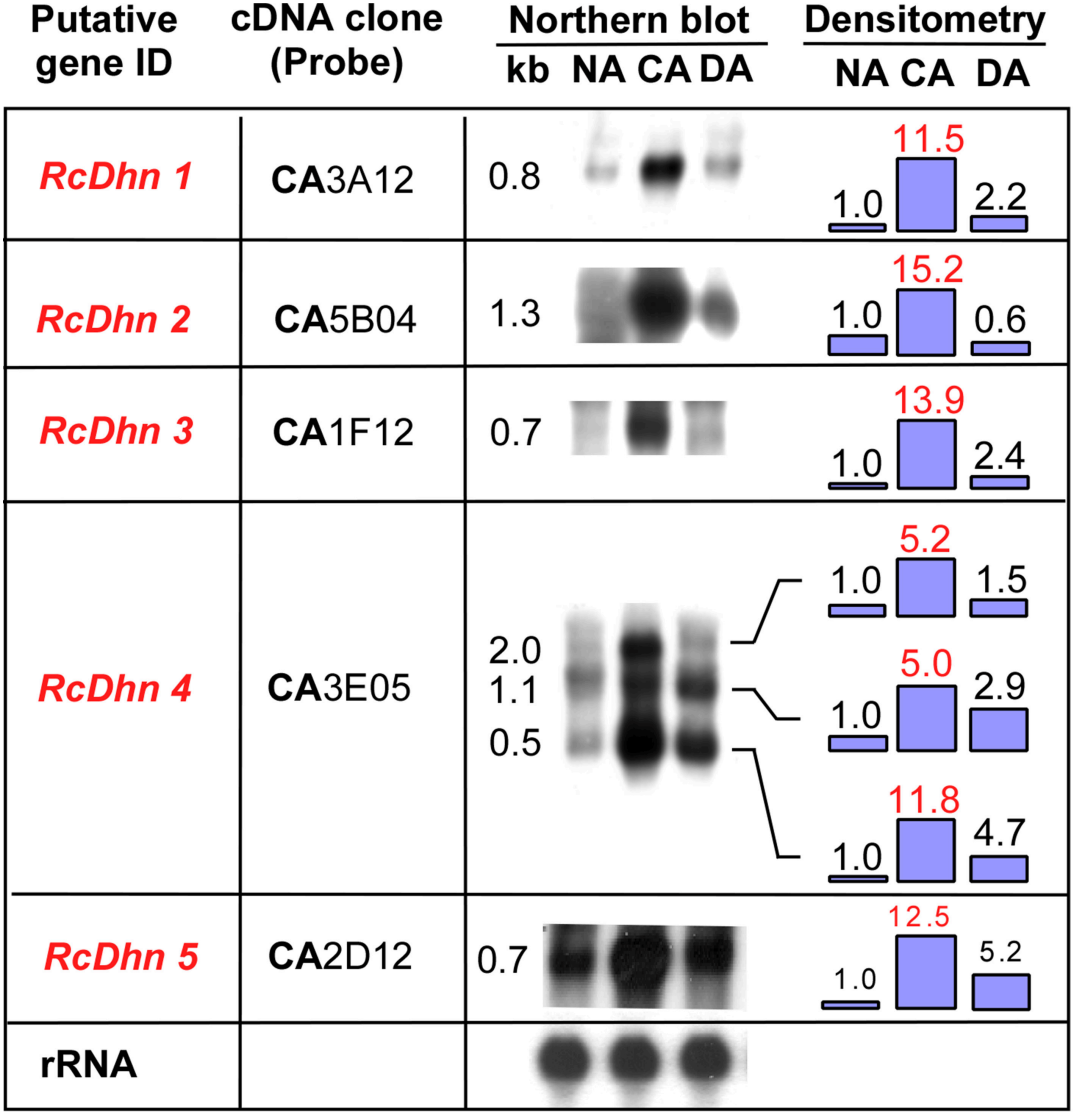
Northern blot analysis for the seasonal expression levels of *RcDhn 1-5* in NA, CA, and DA leaf tissues. Total RNA (8 μg) was isolated and hybridized with respective cDNA probes. Lower panel shows control hybridization of the filters to rRNA using a blueberry cDNA probe confirming equal loadings among the lanes. Fold change in the expression level during cold acclimation and deacclimation relative to non-acclimated state (defined as ‘1') was estimated by densitometry. The putative genes in red text showed at least 5-fold differences in the intensity of bands of northern blot between NA and CA, thus defined as highly cold-responsive genes. *RcDhn 5*'s northern blot data were adapted from a previous study (Peng et al., 2008). CA, cold-acclimated; DA, de-acclimated; NA, non-acclimated.

To further investigate seasonal changes of *RcDhn*s during NA-CA-DA seasonal cycle, we used the respective gene probes to hybridize with the RNA extracted from the non-acclimated (NA), cold acclimated (CA), and deacclimated (DA) *Rhododendron* leaf tissues as described in the Materials and Methods. The northern blot results showed that the transcript levels of all five *RcDhns* followed a distinct seasonal cycle, i.e., relatively low levels in less-hardy tissues in summer followed by ~5–14-fold accumulation (densitometric analysis) in much cold-hardier tissues in winter and then substantial decline in concert with a seasonal transition to spring with the expected loss of freezing tolerance (Figure 4; Peng et al., 2008). The magnitudes of fold-change of these genes among NA CA, and DA tissues indicate that they were all cold-responsive dehydrin genes.

It is noteworthy that northern blot analysis using *RcDhn 4* EST probe revealed three hybridizing mRNA bands of 2.0, 1.1, and 0.5 kb (Figure 4). The predominant band was 0.5 kb, corresponding to the expected size of the *RcDhn 4* EST, which had been previously deposited to GenBank by our group (accession no. CV015159, with mRNA length 491 bp) (Wei et al., 2005a). This *RcDhn 4* EST sequence was used to design primers (as listed in Table 1) for real-time RT-PCR analysis for its monthly expression profiling as described below. The occurrence of three bands on northern blot of *RcDhn 4* indicates the presence of three mature *RcDhn 4* transcripts, which could arise by either alternative splicing, and/or due to alternative transcription initiation or polyadenylation sites.

### Monthly Expression Profiling of *RcDhns* by RT-PCR: Approach II to Identify Cold Acclimation-Responsive Dehydrin Genes

The very hardy species, *R. catawbiense*, has the remarkable ability to increase their leaf freezing tolerance to cope with cold winters (Wei et al., 2005a; Wang et al., 2009). This study examined the monthly gene expression of five *RcDhns* using the samples collected monthly between August (summer) and January (winter); as well as late February. This work provides cold-acclimation-responsive dehydrin gene expression patterns with greater resolution than before, showing that leaf tissues progressively increase their freezing tolerance from summer through fall (Peng et al., 2008). Gene expression profiling was conducted using both regular RT-PCR and real-time RT-PCR. The relative expression levels in each cDNA sample were normalized by comparing the data to the reference gene (e.g., ubiquitin-like protein) in the same sample, which remained constant throughout the season changes. The threshold cycle (Ct) values and calculation worksheets for the fold changes of dehydrin gene expression are provided in Additional File 2, following the data presentation examples for semi-quantitative RT-PCR published in recent literature (Nguyen et al., 2014; Xiong et al., 2016; Feng et al., 2017; Sharma et al., 2017). The statistical analysis process and results are also included in the worksheets (Additional File 2).

**Figure 5:**
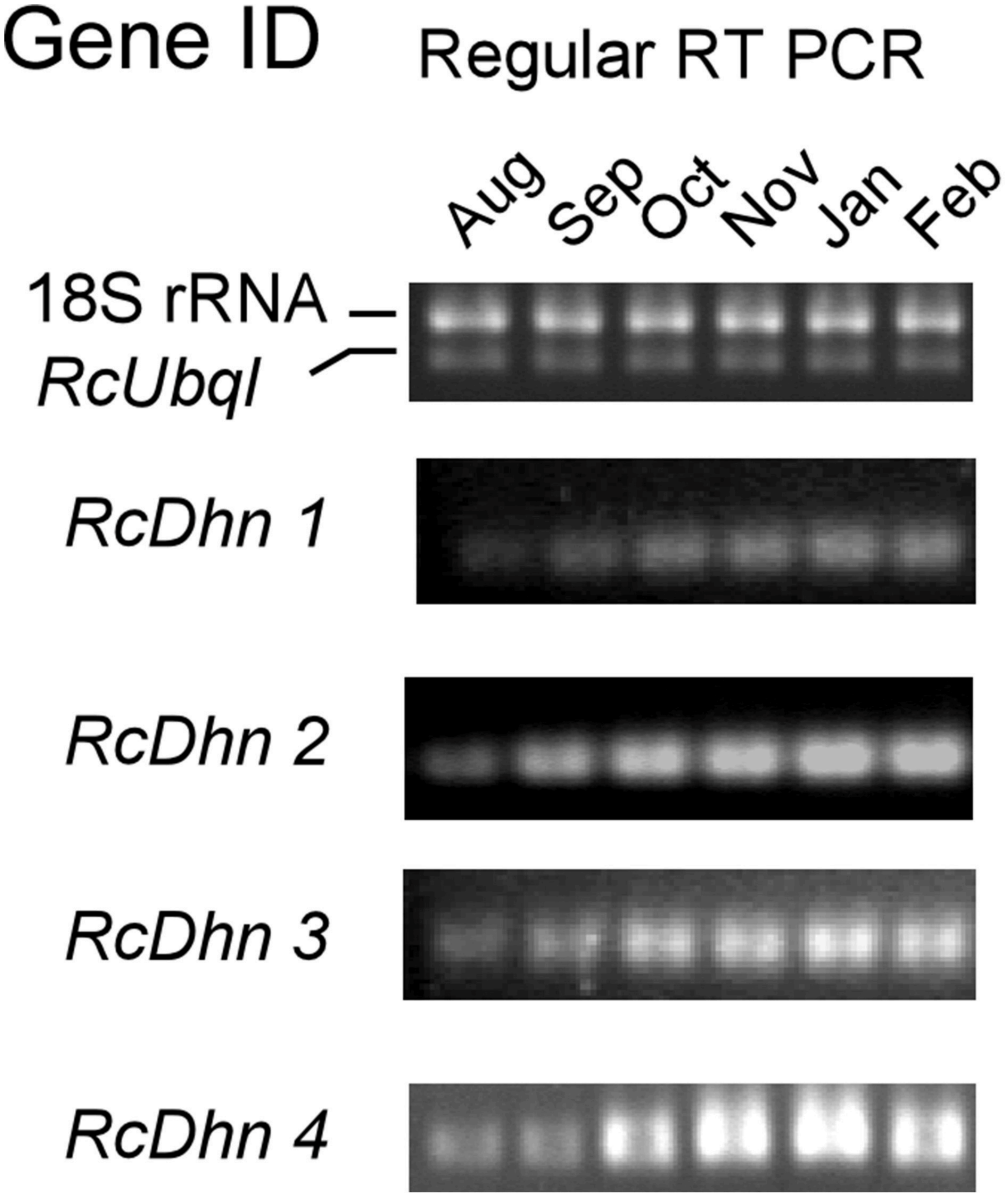
Regular RT-PCR DNA gel images for monthly expression patterns of dehydrin genes in leaf tissues of field-grown *R. catawbiense* collected in August, September, October, November, January, and February. Total RNA extracted from leaf tissues were used for cDNA synthesis. Regular RT-PCR was conducted by using each dehydrin's primers, while the Universal 18S Internal Standards primers (Ambion) and gene-specific primers of rhododendron ubiquitin-like (*RcUbql*) gene were used as the mixed primers for the reference genes; the cycle numbers were 32, which was in the exponential phase of the PCR amplification.

**Figure 6:**
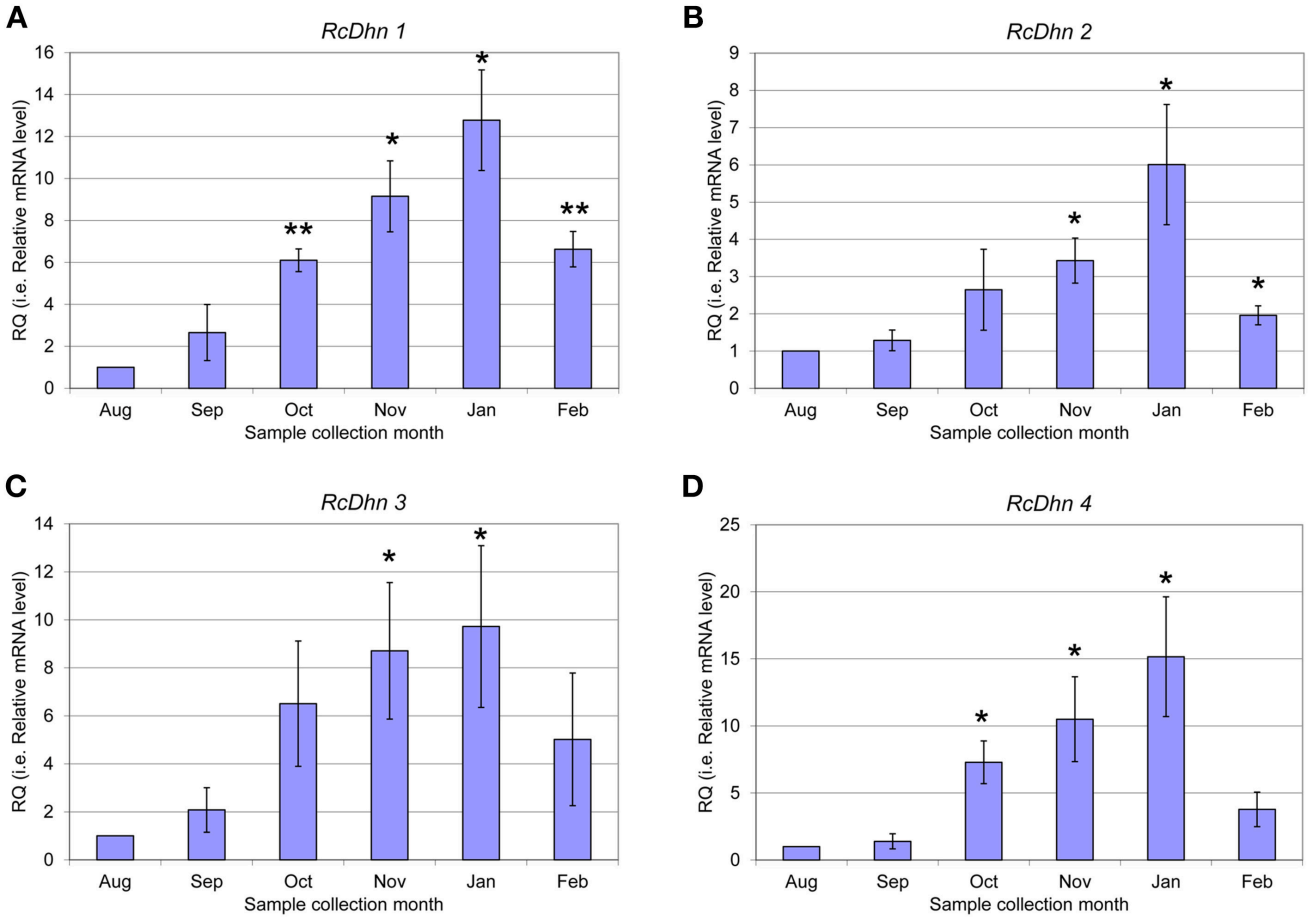
Real-time RT-PCR analysis for monthly expression patterns of dehydrin genes in leaf tissues of field-grown *R. catawbiense* collected in August, September, October, November, January, and February **(A-D)** *RcDhn 1-4*. Total RNA extracted from leaf tissues were used for cDNA synthesis. Real-time RT-PCR was conducted by using each dehydrin's primers, while the gene-specific primers of rhododendron ubiquitin-like (*RcUbql*) gene for the reference gene. The monthly expression of each dehydrin gene was presented as the expression levels relative to its expression level in August (which was set at 1). ^*^ and ^**^ indicate statistical significance of *p* < 0.05 and *p* < 0.01, respectively, comparing with the expression level in August.

The transcript levels of *RcDhns 1, 2, 3, 4*, and *5* followed an incremental accumulation pattern from late August (summer), through October (autumn) till January (winter). The accumulation of transcript began in early autumn (October), and reached a peak in January (Figures 5, 6). Such accumulation pattern mirrors the monthly increase in leaf freezing tolerance from August through January in this species (Peng et al., 2008). Overall, the magnitude of changes of *RcDhns 1-5* genes in *R. catawbiense* between August and January, estimated by real-time RT-PCR, were in the range of 6- to 15-fold, confirming the results observed in the above Approach I for gene expression profiling and supported that they were all cold-acclimation-response dehydrins, which was also similar to the seasonal expression pattern of *RcDhn* 5 previously reported by our group (Peng et al., 2008).

### Orthologous Proteins of Cold-Responsive RcDhn 2 and Distribution of Expanded F-Segment in Protein Database

**Figure 7:**
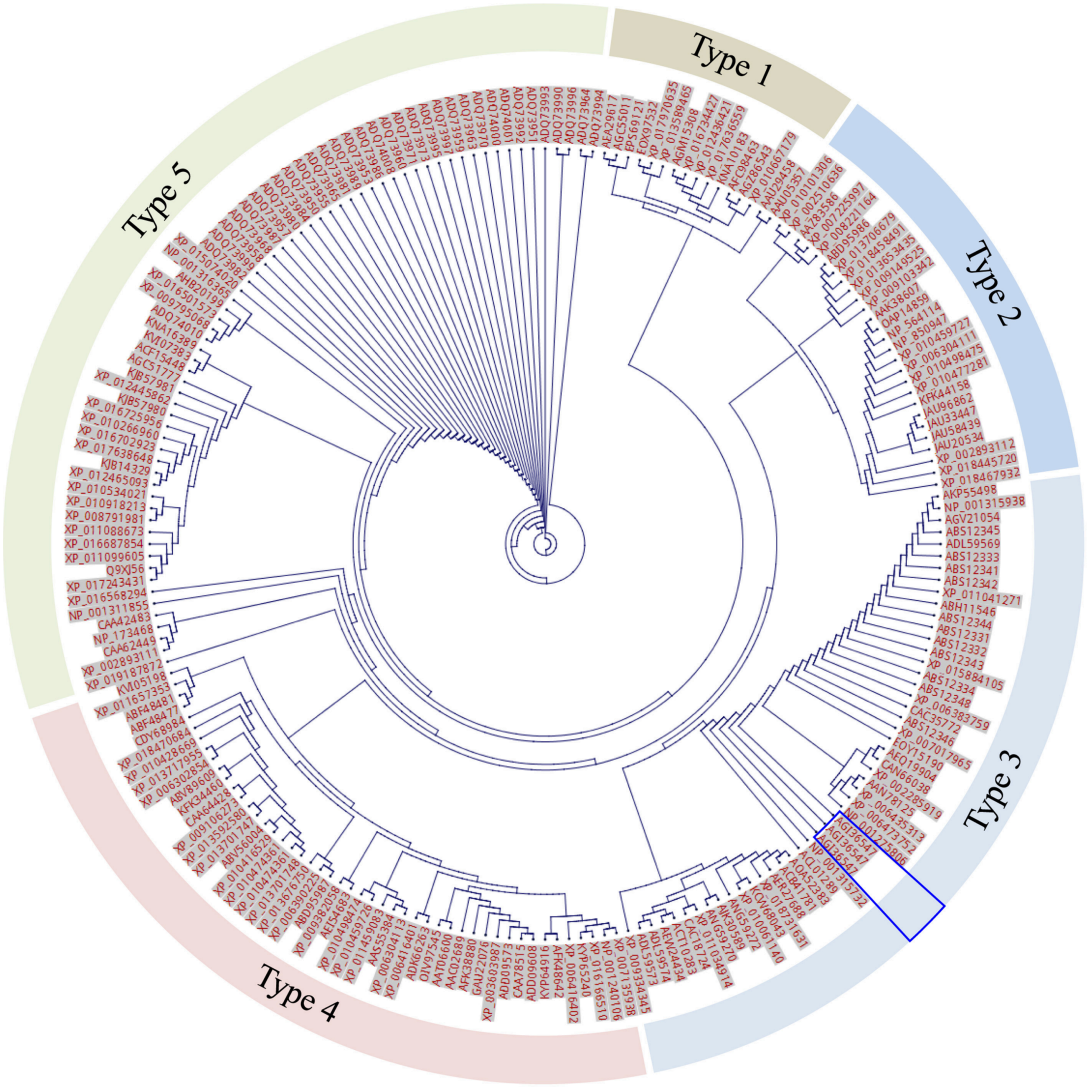
Unrooted tree of total 208 expanded F-segment sequences using CLC Genomics Workbench. The labels beside each dot are protein NCBI accession numbers. The segments are arranged into 5 classes based on the genetic distance. The unique expanded F-segment (three copies from RcDhn 2) is highlighted by the blue box. All are eukaryotes.

To investigate if an expanded F-segment found in RcDhn 2 is conserved in amino acid sequences relative to other dehydrins from other plant species, a bioinformatic study was conducted by two rounds of BlastP search as described in the Materials and Methods and outlined in Figure 2. Briefly, the first round of search was conducted by using the RcDhn 2 amino acid sequence to search for other similar dehydrins using the local BlastP program against the non-redundant protein database (NR), which generated 270 hits. The second round of local BlastP was performed to search for the expanded F-segment consensus sequence using the expanded F-segment initially identified in RcDhn 2 (ETKDRGLFDFLGKKEEEE) as the query and the 270 hits as the database, which led to 208 hits containing the expanded F-segments. Accordingly, the start and end positions of the 208 expanded F-segments in the BlastP result was extracted, and the NCBI accession numbers of resultant hit protein sequences and their contained expanded F-segment sequences are listed in Supplemental Table S1. These 208 expanded F-segment sequences were used to generate the unrooted evolutionary tree using CLC Genomics Workbench 9. Expanded F-segments can be arranged into classes 1, 2, 3, 4, and 5, among which the expanded F-segment in RcDhn 2 belongs to class 3 based on sequence similarity, as illustrated in Figure 7.

**Figure 8:**
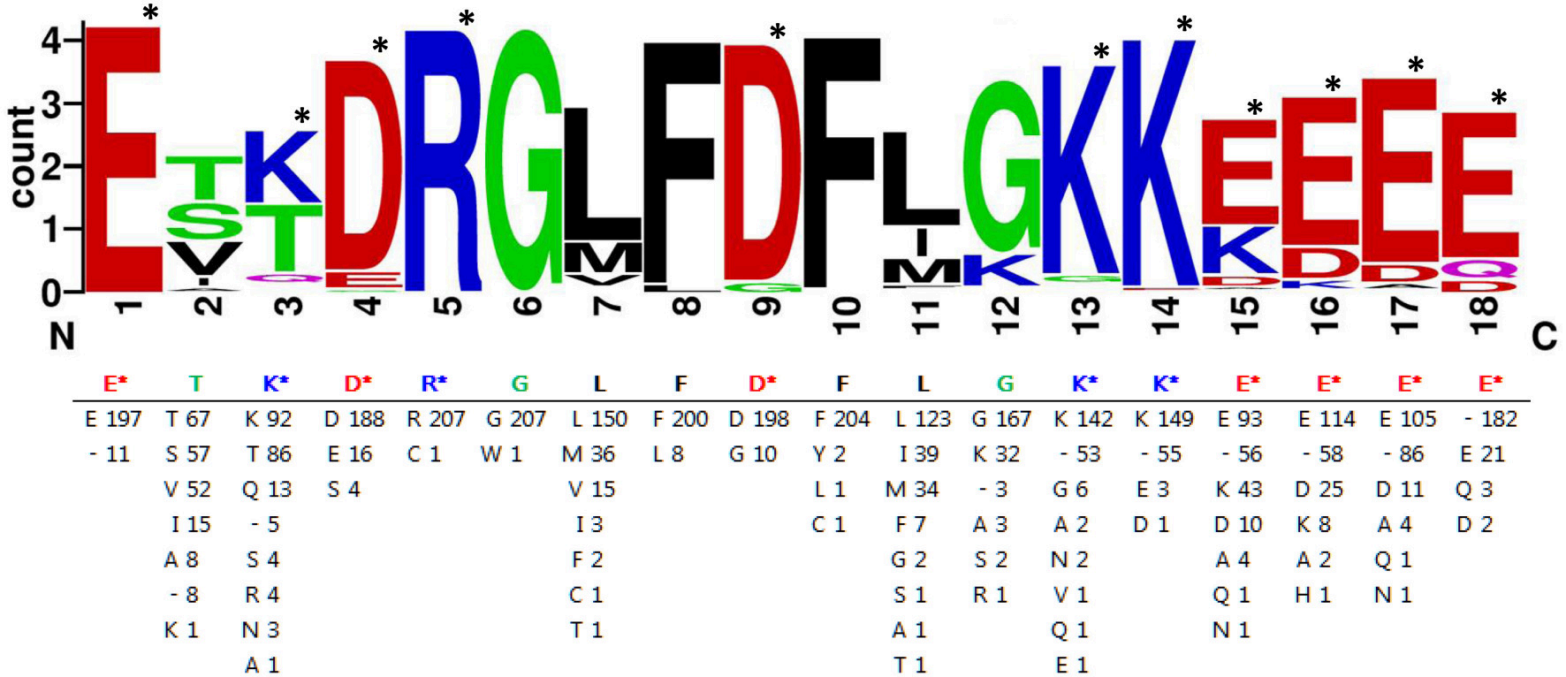
Consensus amino acids of expanded F-segment in dehydrin protein hits from BLASTP search against rhododendron expanded F-segment containing RcDhn2. The sequence graphics in the upper panel was created using WebLogo program. Single letters are abbreviations for amino acids. The number after the letters in the lower panel indicates the occurrence of each amino acid residue at each position. “-” represents a gap in alignment. The most hydrophilic amino acids lysine (K), glutamate (E), aspirate (D) and arginine (R) are indicated by the star marks (^*^), and counted as K_3_E_5_D_2_R_1_.

Furthermore, the 208 expanded F-segment sequences listed in Supplemental Table S1 were used to generate the expanded F-segment consensus sequence, which is presented in two forms. The first form is the WebLogo graphic form (Figure 8, upper panel), which reveals the consensus sequence with a stack of amino acid letters, with the height of each letter representing the observed frequency of the corresponding amino acid at each position. The second form is the conventional form, which is illustrated in Figure 8, lower panel. Whereas, the original expanded F-segment identified in RcDhn 2 is ETKDRGLFDFLGKKEEEE, the expanded F-segment consensus sequence generated from 208 F-segment sequences is E_197_T_67_K_92_D_188_R_207_G_207_L_150_F_200_D_198_F_204_L_123_G_167_K_142_K_149_E_93_E_114_E_105_.

### Comparison of the Expanded F-Segment With K-Segment

As listed in the Supplemental Table S1, the 208 expanded F-segment containing dehydrins were found to exist broadly across a range of species.

**Figure 9:**
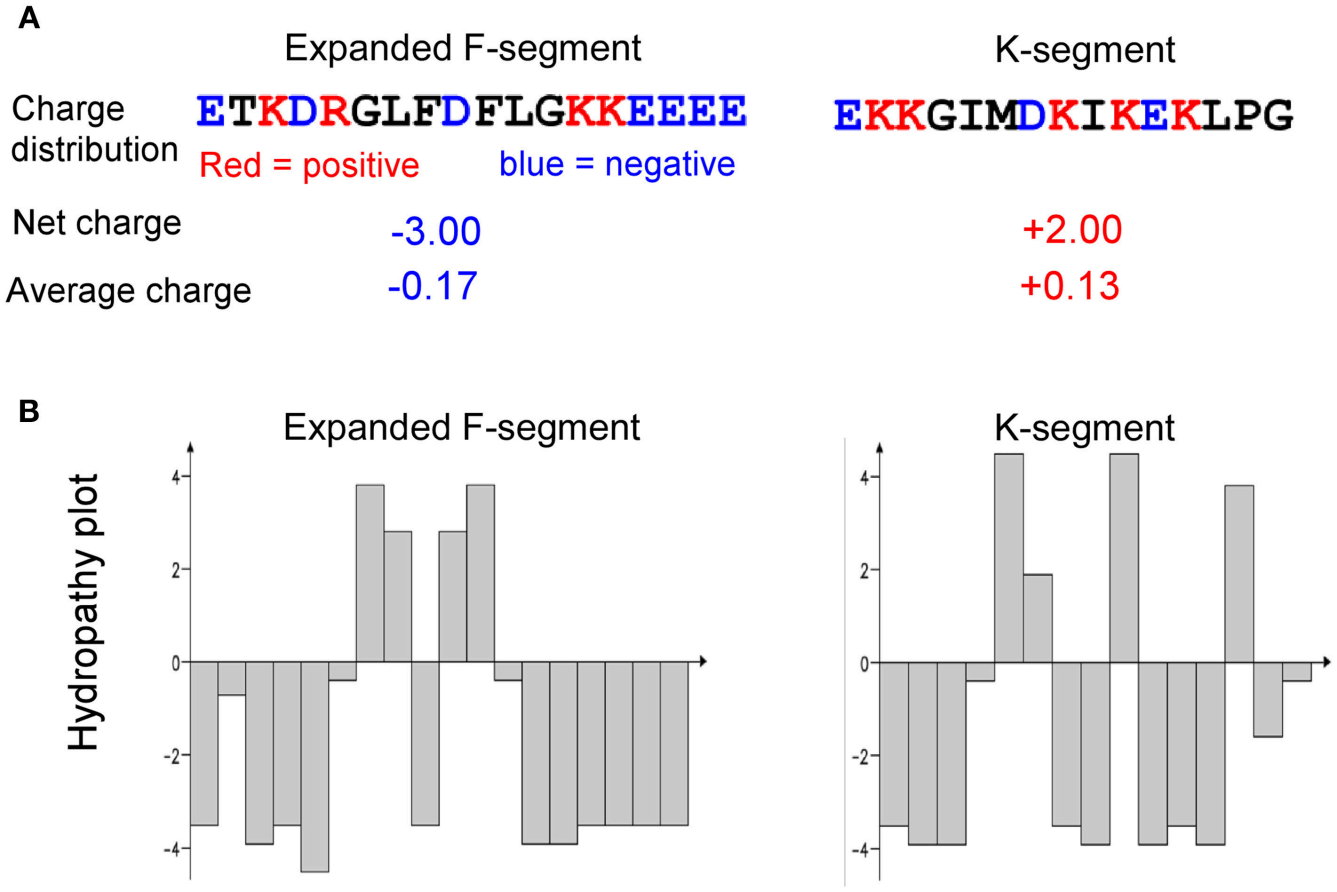
Comparison of charge and hydropathy between the expanded F-segment and K-segment. **(A)** Charge profiling. **(B)** Hydropathy plot. K-segment was reported in literature as described in the text.

Bioinformatics analyses of charge and hydropathy were conducted for the expanded F-segment and the K-segment, the signature sequence of all dehydrins (Campbell and Close, 1997). The results show that the expanded F-segment, explored in this study, contains K_3_E_5_D_2_R_1_; whereas the K-segment consensus contains K_5_E_2_D_1_, as illustrated in Figure 9. It is interesting to note that among the four most hydrophilic amino acids—glutamic acid (E), glutamine (Q), aspartic acid (D) and asparagine (N)—two of them (E and D) are present in the expanded F-segment and the K-segment, which lead to the overall hydrophilic nature of these two motifs. For these two motifs, the expanded F-segment has a more negative net charge (with a value of−3.0) (Figure 9A).

The hydropathy plots are shown in Figure 9B, in which the Kyte–Doolittle scale (Kyte and Doolittle, 1982) was used to compare hydrophobicity by which a positive score indicating hydrophobic and negative score indicating hydrophilic residues. The expanded F-segment has an unique hydropathy plot, which is significantly different from the K-segment (Figure 9B) and may have an implication for its role in interacting with other molecules and subcellular structures, differentiating it from the K-segment motif, as further explored below.

## Discussion

### Possible Function for F-Segment Domain in Dehydrins

Although there is a general acceptance about a broad range of functions of dehydrins, a question still arises as to the fundamental biochemical role of F-segments in dehydrins. Strimbeck (2017) proposed that the F segment may form a short, amphipathic helix capable of binding with membranes or proteins (Strimbeck, 2017). Further biochemical characterization of the F-segments will provide more clues to its structural and functional roles, if any.

It is known that a group of calcium-binding proteins, including calreticulin, calsequestrin, calnexin, and calmegin, use the negatively charged, acidic amino acid region near the C-terminus to bind calcium at high capacity and low affinity (Corbett and Michalak, 2000; Alsheikh et al., 2003). Similarly, an acidic *Arabidopsis* dehydrin was also found to have the ion binding properties (Alsheikh et al., 2003). As described above, the expanded F-segment consensus contains K_3_E_5_D_2_R_1_, and has the lowest net charge (with a value of – 3.0) among three compared motifs. Not surprisingly, the expanded F-segment containing RcDhn 2 is an acidic dehydrin, with the lowest pI of all five RcDhns (a value of 4.8), and it is reasonable to speculate that RcDhn 2 and its orthologs in other species are also likely able to bind with ions, thus may play a role in water retention and/or directly replacing water for the “solvation” of the membrane.

### Implications for the Genetic Engineering of Plants

Dehydrins are a group of intrinsically disordered proteins (lacking secondary and tertiary structure) (Graether and Boddington, 2014) with multiple potential roles, such as cryoprotection, antifreeze proteins (Wisniewski et al., 1999; Reyes et al., 2008), metal binding/ion sequestration, antioxidants (Svensson et al., 2000; Alsheikh et al., 2003; Hara et al., 2005, 2013), and chaperone properties (Kovacs et al., 2008). Current study identified five *RcDhn* genes related to stress tolerance traits. These can be used for genetic engineering of plants to enhance their cold adaptation capacity. A previous study conducted by co-authors and collaborators supports the feasibility of this approach, demonstrating that *Arabidopsis* plants overexpressing RcDhn 5 were significantly more freeze-tolerant than the wild-type controls (Peng et al., 2008); same dehydrin also was shown to provide cryoprotection and dehydration-stress tolerance, *in vitro*, to cold labile lactate dehydrogenase protein (Peng et al., 2008; Reyes et al., 2008).

Based on the amino acid sequence data (Figure 3), we propose here that RcDhn 5 may also have a metal/ion-binding property. This SK2-type acidic dehydrin contains a histidine-rich sequence. Published research suggests that metal/ion binding property may be restricted to acidic, SK-type dehydrins (Alsheikh et al., 2005) and that histidine-rich motif is characteristic of metal binding proteins (Hernández-Sánchez et al., 2014). Hernández-Sánchez et al. (2015) reported that the deletion of histidine-rich motif in cactus dehydrin OpsDHN1 restricted its localization to cytoplasm, and the deletion of its S-segment also affected its nuclear localization (Hernández-Sánchez et al., 2015). We speculate that the histidine-rich motif of RcDhn5 may also be involved in the similar function as both histidine- and serine-rich motifs exist in RcDhn5, and further studies are needed to test this proposal.

It has also been shown for several SK-type dehydrins (ERD14, ERD10, and COR47 of *A. thaliana*) that activation of their ion-binding (Ca^2+^−binding) property may require phosphorylation and that this phosphorylation site is contained within the serine (S) motif (Alsheikh et al., 2005). Presence of a serine tract in RcDhn 5 sequence (Figure 3) is in line with this proposition.

In addition, the transcript levels of all five RcDhns genes increased throughout the autumn and reached a peak in the middle of winter season (January), as illustrated in Figures 5, 6. This prompts us to further propose that the sequences of *RcDhns* promoters can be explored for the temporal control of expressing heterologous cold-hardiness related genes aiming to enhance cold acclimation of plants. Literature shows that promoters selected from highly expressed genes are effective to build expression vector for expressing heterologous genes in eukaryotic organisms (Poulsen et al., 2006).

### Future Studies for Gene Structure Analysis of *RcDhn4* and Its Variants

It is interesting that we identified three mature transcripts of the *RcDhn 4* gene, which could arise by alternative splicing, and alternative transcription initiation or polyadenylation sites. Furthermore, it is noteworthy that SKn-type dehydrins are known to typically contain one intron sequence within the S-segment (Jiménez-Bremont et al., 2013). It would be interesting to determine, by obtaining and comparing the genomic sequence with the cDNA sequence of *RcDhn 4* gene, whether this gene also contains intron(s). If the presence of intron is indeed confirmed, it can be used to design the intron-flanking PCR molecular markers for rhododendron genetic mapping, since it is assumed that intronic regions have richer polymorphism than exonic regions (Wei et al., 2005b).

## Conclusion

Multiple approaches were taken to identify and characterize five RcDhns and examine their transcriptional profiling over the course of NA, CA and DA spanning from summer, autumn, winter, and spring. Their transcript expression patterns indicated that *RcDhn 1-5* had 5- to 10-fold upregulation during the cold acclimation process, followed by a significant downregulation in spring as plants lose their previously acquired freezing tolerance, supporting the roles of these cold-responsive genes in plant freezing tolerance. The identification of an unique expanded F-segment consensus sequence in RcDhn 2 and its orthologs across a broad range of species, together with their negative charge and hydrophilic nature, highlight their potential to be used for genetic engineering of crops and bioenergy plants for improved cold tolerance.

## Author Contributions

RA and HW lead the project and coordinated the study, conceived designed and executed the experiments related to the transcriptional analysis. S-YD designed and YY executed the bioinformatic analysis. HW coordinated the manuscript preparation. All authors contributed to the data analyses, read, revised and approved the final manuscript.

### Conflict of Interest Statement

The authors declare that the research was conducted in the absence of any commercial or financial relationships that could be construed as a potential conflict of interest.

## Supplementary Material

### Additional File 1

Supplemental Figure S1 Diagram showing four BlastP search hits that were not included in the consensus sequence analysis of the F-segment.

Supplemental Table S1 List of 208 putative proteins identified as the orthologs for the expanded F–segment containing RcDhn 2.

### Additional File 2

Threshold cycle (Ct) values and fold change calculation worksheets for the monthly expression of rhododendron dehydrin genes.

## Abbreviations

ABA: abscisic acid
CA: cold-acclimated
DA: de-acclimated
Dhn: dehydrin
EST: expressed sequence tag
NA: non-acclimated
RcDhn: *Rhododendron catawbiense* dehydrin.
