# Supplementary material for: Identification and Characterization of Five Cold Stress-Related Rhododendron Dehydrin Genes: Spotlight on a FSK-Type Dehydrin With Multiple F-Segments
Source: Front Bioeng Biotechnol. 2019 Feb 21;7:30. doi: 10.3389/fbioe.2019.00030 (PMC6393390; doi:10.3389/fbioe.2019.00030)
Supplement: Supplemental Figure S1 — Diagram showing four BlastP search hits that were not included in the consensus sequence analysis of the F-segment. [file Table_1.DOCX]

Identification and characterization of five cold stress-related rhododendron dehydrin genes: spotlight on a FSK-type dehydrin with multiple F-segments

**Additional file 1:**

**File type:** .docx

**List of contents:**

- **Supplemental Figure S1**. Diagram showing four BlastP search hits that were not included in the consensus sequence analysis of the F segment.
- **Supplemental Table S1.** List of 208 putative proteins identified as the orthologs for the expanded F-segment containing RcDhn 2.


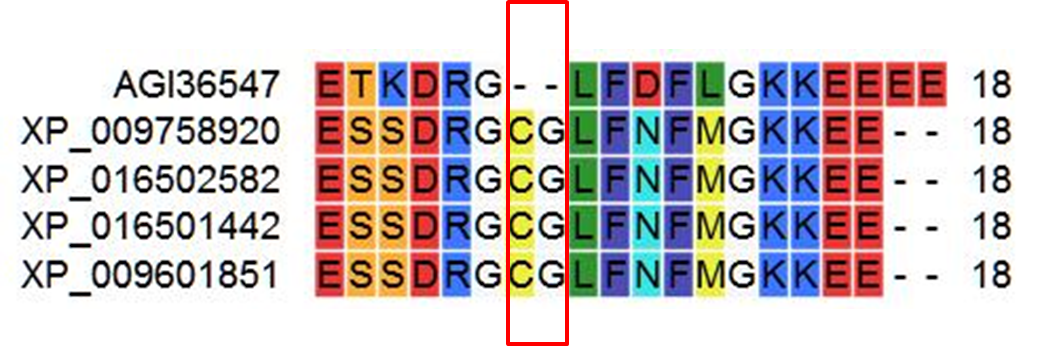


**Supplemental Figure S1. Diagram showing four BlastP search hits that were not included in the consensus sequence analysis of the F segment.** The two insert amino acids (CG), which are in the red box in four XP segments, affect the count of the alignment result, thus were removed during alignment.

**Supplemental Table S1. List of 208 putative proteins identified as the orthologs for the expanded F-segment containing RcDhn 2.** The table is sorted in the same order of accession numbers within Figure 7.

| **Row no.** | **NCBI Accession no.** | **E-segment sequence** | **Protein Name** | **Species** |
| --- | --- | --- | --- | --- |
| **Type 1** |  |  |  |  |
| 1 | AEA29617 | ETTDRG--LFDFM--KKKEDE | dehydrin | *Suaeda glauca* |
| 2 | AGC55011 | ETTDRG--LFDFM--KKKEDE | dehydrin | *Suaeda salsa* |
| 3 | EPS69121 | ESTDRG--LFDFAKGKKDE-- | hypothetical protein M569 05646, partial | *Genlisea aurea* |
| 4 | EOX97532 | ETTDRG--MFDFL--KEDEDD | Uncharacterized protein TCM 006526 | *Theobroma cacao* |
| 5 | XP_017970635 | ETTDRG--MFDFL--KKEDED | phosphoprotein ECPP44 | *Theobroma cacao* |
| 6 | XP_013589465 | EVTDRG--MFDFL---KKKNE | dehydrin ERD14-like | *Brassica oleracea var. oleracea* |
| 7 | AGM15308 | ETTDRG--MFDFMK-KKD--- | dehydrin 1 | *Chenopodium quinoa* |
| 8 | XP_016734427 | ETTDRG--MFDFMK-KKDEEQ | phosphoprotein ECPP44-like | *Gossypium hirsutum* |
| 9 | XP_012436421 | ETTDRG--MFDFMK-KKDEEQ | phosphoprotein ECPP44-like | *Gossypium raimondii* |
| 10 | XP_017636559 | ETTDRG--MFDFMK-KKDEEQ | phosphoprotein ECPP44-like | *Gossypium arboreum* |
| 11 | KNA10183 | ETTDRG--LFDFM--KKKD-- | hypothetical protein SOVF 146760 | *Spinacia oleracea* |
| 12 | AFC98463 | ESTDRG--MFDFLK-KKDHAE | dehydrin | *Atriplex canescens* |
| 13 | AGZ86543 | ESTDRG--MFDFMK-KKDDAE | dehydrin | *Atriplex halimus* |
| 14 | XP_010667179 | ESTDRG--LFDFMK-KKDD-D | phosphoprotein ECPP44 | *Beta vulgaris subsp. vulgaris* |
| 15 | AAU29458 | ESTDRG--LFGF--GAKKEEE | dehydrin, partial | *Salvia miltiorrhiza* |
| 16 | AAU05357 | ESTDRG--LFGF--GAKKEEE | dehydration protein, partial | *Salvia miltiorrhiza* |
| **Type 2** |  |  |  |  |
| 17 | XP_010101306 | ESKDRG--LFDFL-------- | Dehydrin ERD10 | *Morus notabilis* |
| 18 | XP_002510636 | ESKDRG--LFDFLG------- | phosphoprotein ECPP44 | *Ricinus communis* |
| 19 | AAZ83586 | EIKDRG--LFDFL-GKK-EAE | type II SK2 dehydrin, partial | *Prunus persica* |
| 20 | XP_007225997 | EIKDRG--LFDFL-GKK-EAE | hypothetical protein PRUPE ppa010326mg | *Prunus persica* |
| 21 | XP_008221164 | EIKDRG--LFDFL-GKK---- | dehydrin COR47-like | *Prunus mume* |
| 22 | ABD95986 | EVKDRG--LFDFL-GKK-EE- | dehydrin, partial | *Brassica juncea* |
| 23 | XP_013706679 | EVKDRG--LFDFL-GKK-EE- | dehydrin ERD10-like | *Brassica napus* |
| 24 | XP_018458491 | EVKDRG--LFDFL-GKK-EE- | dehydrin ERD10-like | *Raphanus sativus* |
| 25 | XP_013653435 | EVKDRG--LFDFL-GKK-EE- | dehydrin ERD10-like | *Brassica napus* |
| 26 | XP_009149525 | EVKDRG--LFDFL-GKK-EE- | dehydrin ERD10-like | *Brassica rapa* |
| 27 | XP_009103342 | EVKDRG--LFDFL-GKK-EE- | dehydrin ERD10-like | *Brassica rapa* |
| 28 | AAK38607 | EIKERG--MFDFL---KKKEE | unknown protein | *Arabidopsis thaliana* |
| 29 | OAP14859 | EIKERG--MFDFL---KKKEE | LTI45 | *Arabidopsis thaliana* |
| 30 | NP_564114 | EIKERG--MFDFL---KKKEE | Dehydrin family protein | *Arabidopsis thaliana* |
| 31 | NP_850947 | EIKERG--MFDFL---KKKEE | Dehydrin family protein | *Arabidopsis thaliana* |
| 32 | XP_010459727 | EIKERG--MFDFL---KKKEE | dehydrin ERD10-like | *Camelina sativa* |
| 33 | XP_006304111 | EIKERG--MFDFL---KKKEE | hypothetical protein CARUB v10010037mg | *Capsella rubella* |
| 34 | XP_010498475 | --KERG--MFDFL---KKKEE | dehydrin ERD10-like | *Camelina sativa* |
| 35 | XP_010477281 | --KERG--MFDFL---KKKEE | dehydrin ERD10-like | *Camelina sativa* |
| 36 | KFK44158 | EVKERG--MFDFL-KKKEE-- | hypothetical protein AALP AA1G222500 | *Arabis alpina* |
| 37 | JAU96862 | EVKSRG--MFDFL-KKKEE-- | Dehydrin ERD10, partial | *Noccaea caerulescens* |
| 38 | JAU33447 | EVKSRG--MFDFL-KKKEE-- | Dehydrin ERD10, partial | *Noccaea caerulescens* |
| 39 | JAU58439 | EVKSRG--MFDFL-KKKEE-- | Dehydrin ERD10 | *Noccaea caerulescens* |
| 40 | JAU20534 | EVKSRG--MFDFL-KKKEE-- | Dehydrin ERD10 | *Noccaea caerulescens* |
| 41 | XP_002893112 | EVKERG--LFDFL-KKKEE-- | hypothetical protein ARALYDRAFT 472273 | *Arabidopsis lyrata subsp. lyrata* |
| 42 | XP_018445720 | EVKDRG--LFDFL-KKKEE-- | dehydrin ERD10-like | *Raphanus sativus* |
| 43 | XP_018467932 | EAKDRG--LFDFL-KKKEE-- | dehydrin ERD10-like | *Raphanus sativus* |
| **Type 3** |  |  |  |  |
| 44 | AKP55498 | ETKDRG--LFDFL-GKKAE-- | dehydrin 3, partial | *Pyrus pyrifolia* |
| 45 | NP_001315938 | ETKDRG--LFDFL-GKKAEE- | dehydrin COR47-like | *Malus domestica* |
| 46 | AGV21054 | ETKDRG--LFDFL-GKKAEE- | dehydrin 5 | *Eriobotrya japonica* |
| 47 | ABS12345 | ETKDRG--LFDFL-G------ | dehydrin | *Populus alba* |
| 48 | ADL59569 | ETKDRG--LFDFL-G------ | dehydrin 1 | *Populus alba x Populus glandulosa* |
| 49 | ABS12333 | ETKDRG--LFDFL-G------ | dehydrin | *Populus davidiana* |
| 50 | ABS12341 | ETKDRG--LFDFL-G------ | dehydrin | *Populus davidiana* |
| 51 | ABS12342 | ETKDRG--LFDFL-G------ | dehydrin | *Populus davidiana* |
| 52 | XP_011041271 | ETKDRG--LFDFL-G------ | phosphoprotein ECPP44 | *Populus euphratica* |
| 53 | ABH11546 | ETKDRG--LFDFL-G------ | dehydrin | *Populus alba x Populus glandulosa* |
| 54 | ABS12344 | ETKDRG--LFDFL-G------ | dehydrin | *Populus glandulosa* |
| 55 | ABS12331 | ETKDRG--LFDFL-G------ | dehydrin | *Populus davidiana* |
| 56 | ABS12332 | ETKDRG--LFDFL-G------ | dehydrin | *Populus davidiana* |
| 57 | ABS12343 | ETKDRG--LFDFL-G------ | dehydrin | *Populus davidiana* |
| 58 | XP_015884105 | ETKDRG--LFDFL-G------ | dehydrin ERD14 | *Ziziphus jujuba* |
| 59 | ABS12334 | ETKDRG--LFDFL-G------ | dehydrin | *Populus davidiana* |
| 60 | ABS12348 | ETKDRG--LFDFL-G------ | dehydrin | *Populus x canadensis* |
| 61 | XP_006383759 | ETKDRG--LFDFL-G------ | dehydrin family protein | *Populus trichocarpa* |
| 62 | CAC35772 | ETKDRG--LFDFL-G------ | dhn1, partial | *Populus x canadensis;Populus nigra* |
| 63 | ABS12346 | ETKDRG--LFDFL-G------ | dehydrin | *Populus maximowiczii* |
| 64 | XP_007017965 | ETKDRG--LFDFL-G------ | dehydrin COR47 | *Theobroma cacao* |
| 65 | EOY15190 | ETKDRG--LFDFL-G------ | Dehydrin 2, putative | *Theobroma cacao* |
| 66 | AEQ19904 | ETKDRG--IFDFLG------- | dehydrin 2 | *Vitis yeshanensis* |
| 67 | CAN66038 | ETKDRG--IFDFLG------- | hypothetical protein VITISV 010455 | *Vitis vinifera* |
| 68 | XP_002285919 | ETKDRG--IFDFLG------- | dehydrin ERD14 | *Vitis vinifera* |
| 69 | AAN78125 | ETKDRG--MLDFL-GKKEEE- | dehydrin | *Citrus x paradisi* |
| 70 | XP_006435313 | ETKDRG--MLDFL-GKKEEE- | hypothetical protein CICLE v10002349mg | *Citrus clementina* |
| 71 | XP_006473752 | ETKDRG--MLDFL-GKKEEE- | dehydrin isoform X1 | *Citrus sinensis* |
| 72 | NP_001275806 | ETKDRG--MLDFL-GKKEEE- | dehydrin | *Citrus sinensis* |
| 73 | AGI36547 | ETKDRG--LFDFL-GKKEEEE | dehydrin 2 | *Rhododendron catawbiense* |
| 74 | AGI36547 | ETKDRG--LFDFL-GKKEEEE |  |  |
| 75 | AGI36547 | -TKDRG--LFDFL-GKKEEEE |  |  |
| 76 | NP_001315732 | ETKDRG--LFDFL-GKKEEE- | dehydrin COR47-like | *Malus domestica* |
| 77 | ACL01289 | ETKDRG--LFDFL-GKKEEE- | dehydrin 2 | *Eriobotrya japonica* |
| 78 | AOA52383 | ETQDRG--LFDFM-------- | dehydrin 2 | *Eucalyptus nitens* |
| 79 | ACB41781 | ETADRG--LFDFTAVKQKEE- | dehydrin | *Rhododendron catawbiense* |
| 80 | AER27688 | ETQDRG--LFDFM-------- | dehydrin 2 | *Eucalyptus globulus* |
| 81 | XP_018731631 | ETQDRG--LFDFMG------- | dehydrin ERD14-like | *Eucalyptus grandis* |
| 82 | KCW68043 | ETRDR--WLFDFMG------K | hypothetical protein EUGRSUZ F01726 | *Eucalyptus grandis* |
| 83 | XP_010061140 | ETRDRG--LFDFMG------- | phosphoprotein ECPP44-like | *Eucalyptus grandis* |
| 84 | ANG59272 | ETKDRG--LFDFMG------- | dehydrin 2 | *Hevea brasiliensis* |
| 85 | AJK30589 | ETKDRG--LFGFLG------- | dehydrin | *Hevea brasiliensis* |
| 86 | ANG59270 | ETKDRG--LFGFLG------- | dehydrin 1 | *Hevea brasiliensis* |
| 87 | XP_011034914 | ETKDRG--LFGFM-GKKKEE- | neurofilament heavy polypeptide-like | *Populus euphratica* |
| 88 | CAC18724 | ETKDRG--LFGFM-GKKKEE- | putative dehydrin, partial | *Populus x canadensis* |
| 89 | ACT10283 | ETQDRG--LFGFMG------- | dehydrin | *Camellia sinensis* |
| 90 | AEW24434 | ETQDRG--LFGFMG------- | dehydrin 1 | *Camellia sinensis* |
| 91 | ADL59574 | ETRDRG--LFGFMG------- | dehydrin 7 | *Populus alba x Populus glandulosa* |
| 92 | ADL59573 | ETRDRG--LFGFMG------- | dehydrin 2 | *Populus alba x Populus glandulosa* |
| 93 | XP_009334345 | ETNDRG--LFDFL-GKKAE-- | dehydrin COR47 | *Pyrus x bretschneideri* |
| **Type 4** |  |  |  |  |
| 94 | XP_007135938 | EVQDRG--VFDFLG------- | hypothetical protein PHAVU 009G004400g | *Phaseolus vulgaris* |
| 95 | NP_001240106 | EVQDRG--VFDFL-GKKKDEE | uncharacterized protein LOC100816984 | *Glycine max;Glycine soja* |
| 96 | XP_016166510 | EVQDRG--VLDFL-GKKKE-- | phosphoprotein ECPP44-like | *Arachis ipaensis* |
| 97 | KYP65240 | EVKDRG--VLDFL-GKKKEEE | Phosphoprotein ECPP44 | *Cajanus cajan* |
| 98 | XP_006416402 | EVKDRG--VFDFL-GQKKEE- | hypothetical protein EUTSA v10008706mg | *Eutrema salsugineum* |
| 99 | AFK48642 | EINDRG--VFDFL-GKKKEQD | unknown | *Lotus japonicus* |
| 100 | KYP64916 | EVNDRG--VFDFL-GKKKEE- | Dehydrin COR47 | *Cajanus cajan* |
| 101 | ADD09608 | EIKDRG--VFDFLGGKKKDE- | dehydrin b | *Trifolium repens* |
| 102 | CAA78515 | EIKDRG--VFDFLGGKKKDE- | dehydrin-cognate (macronuclear) | *Pisum sativum* |
| 103 | ADD09573 | EIKDRG--VFDFLGGKKKDE- | dehydrin b | *Trifolium repens* |
| 104 | XP_003603987 | EIKDRG--VFDFLGGKKKDE- | dehydrin | *Medicago truncatula* |
| 105 | GAU22076 | --KDRG--VFDFLGGKKKDE- | hypothetical protein TSUD 309800 | *Trifolium subterraneum* |
| 106 | AFK38880 | ETQDRG--VFDFLG------- | unknown | *Lotus japonicus* |
| 107 | AAC02689 | ETQDRG--TFD-LSGNKKEDE | cold regulated LTCOR18 | *Lavatera thuringiaca* |
| 108 | AAT06600 | EIKDCG--VFDFFGNKKEEE- | dehydrin | *Lupinus albus* |
| 109 | OIV97545 | ETKDRG--VLDFF-GKKKEEE | hypothetical protein TanjilG 12302 | *Lupinus angustifolius* |
| 110 | ADK66263 | EVKDRG--FFDFL-GKKKEE- | dehydrin | *Eutrema salsugineum* |
| 111 | XP_006416401 | EVKDRG--FFDFL-GKKKEE- | hypothetical protein EUTSA v10008313mg | *Eutrema salsugineum* |
| 112 | XP_006304113 | ---DRG--MFDFLSKKKEE-- | hypothetical protein CARUB v10010040mg | *Capsella rubella* |
| 113 | AAS55384 | ---DRG--MFDFLSKKKEE-- | dehydrin cor29 | *Capsella bursa-pastoris* |
| 114 | XP_011459083 | ETQDRG--MFDFL-GKKKEEE | LOW QUALITY PROTEIN: dehydrin COR47-like | *Fragaria vesca subsp. vesca* |
| 115 | XP_010459726 | EVTDRG--MFDFL-GKKKEAE | dehydrin COR47-like | *Camelina sativa* |
| 116 | XP_010498474 | EVTDRG--MFDFL-GKKKEAE | dehydrin COR47-like | *Camelina sativa* |
| 117 | AEI54683 | EVQDRG--LFDFLG------- | SK3-type dehydrin | *Musa ABB Group* |
| 118 | XP_009382058 | EVQDRG--LFDFLG------- | dehydrin COR410 | *Musa acuminata subsp. malaccensis* |
| 119 | ABD95987 | EVTDRG--LFDFL-GKKKE-E | dehydrin | *Brassica juncea* |
| 120 | XP_006390225 | EVTDRG--LFDFL-GKKKE-E | hypothetical protein EUTSA v10019152mg | *Eutrema salsugineum* |
| 121 | XP_013676750 | EVTDRG--LFDFL-G------ | dehydrin ERD14-like | *Brassica napus* |
| 122 | XP_013701748 | EVTDRG--LFDFL-GKKKDE- | dehydrin ERD14-like isoform X2 | *Brassica napus* |
| 123 | XP_010474361 | EVTDRG--LFDFL-GKKKDE- | dehydrin ERD14 | *Camelina sativa* |
| 124 | XP_010474361 | EVTDRG--LFDFL-GKKKDE- |  |  |
| 125 | XP_010416529 | EVTDRG--LFDFL-GKKKDE- | dehydrin ERD14-like | *Camelina sativa* |
| 126 | ABV56004 | EVTDRG--LFDFL-GKKKDE- | dehydrin protein | *Capsella bursa-pastoris* |
| 127 | XP_013701747 | EVTDRG--LFDFL-GKKKDE- | dehydrin ERD14-like isoform X1 | *Brassica napus* |
| 128 | XP_013592580 | EVTDRG--LFDFL-GKKKDE- | dehydrin ERD14 | *Brassica oleracea var. oleracea* |
| 129 | XP_009106273 | EVTDRG--LFDFL-GKKKDE- | dehydrin ERD14 | *Brassica rapa* |
| 130 | CAA64428 | EVTDRG--LFDFL-GKKKDE- | pollen coat protein | *Brassica napus;Brassica oleracea* |
| 131 | KFK34460 | EVTDRG--LFDFL-GKKKDE- | hypothetical protein AALP AA5G148200 | *Arabis alpina* |
| 132 | ABV89609 | EVTDRG--LFDFL-GKKKDE- | pollen coat protein | *Brassica rapa* |
| 133 | XP_006302854 | EVTDRG--LFDFL-GKKKDE- | hypothetical protein CARUB v10020984mg | *Capsella rubella* |
| 134 | XP_013717955 | EVTDRG--LFDFL-GKKKDE- | dehydrin ERD14-like | *Brassica napus* |
| 135 | XP_010428669 | EVTDRG--LFDFF-GKKKDE- | dehydrin ERD14-like | *Camelina sativa* |
| 136 | XP_018470684 | EVTDRG--LFDFL-G------ | dehydrin ERD14-like | *Raphanus sativus* |
| 137 | CDY68984 | EVTDRG--LFDFL-G------ | BnaAnng29030D | *Brassica napus* |
| 138 | ABF48477 | ETTDRG--LFDFLGAKKDE-- | dehydrin 4 | *Panax ginseng* |
| 139 | ABF48481 | ETTDRG--LFDFLGAKKDE-- | dehydrin 8 | *Panax ginseng* |
| 140 | XP_011657353 | EATDRG--LFDFLG------- | phosphoprotein ECPP44 | *Cucumis sativus* |
| 141 | KVI05198 | ---DRG--LFDFLG------- | Dehydrin | *Cynara cardunculus var. scolymus* |
| 142 | XP_019187872 | ---DRG--LLDFL-GKKEEE- | phosphoprotein ECPP44-like | *Ipomoea nil* |
| **Type 5** |  |  |  |  |
| 143 | XP_002893111 | EVTDRG--LFDFL-GKKEE-E | hypothetical protein ARALYDRAFT 889495 | *Arabidopsis lyrata subsp. lyrata* |
| 144 | CAA62449 | EVTDRG--LFDFL-GKKEE-E | dehydrin | *Arabidopsis thaliana* |
| 145 | NP_173468 | EVTDRG--LFDFL-GKKEE-E | cold-regulated 47 | *Arabidopsis thaliana* |
| 146 | CAA42483 | EVTDRG--LFDFL-GKKEE-E | cor47, partial | *Arabidopsis thaliana* |
| 147 | NP_001311855 | ESTDRG--LFDFL-GKKEEE- | phosphoprotein ECPP44-like | *Capsicum annuum* |
| 148 | XP_016568294 | ESTDRG--LFDFL-GKKEEE- | phosphoprotein ECPP44-like | *Capsicum annuum* |
| 149 | XP_017243431 | ESSDRG--LFDFMK-KEEKDE | phosphoprotein ECPP44 | *Daucus carota subsp. sativus* |
| 150 | Q9XJ56 | ESSDRG--LFDFMK-KEEKDE | RecName: Full=Phosphoprotein ECPP44 | *Daucus carota* |
| 151 | XP_011099605 | ESSDRG--LFDFMG------- | dehydrin ERD14-like | *Sesamum indicum* |
| 152 | XP_016687854 | ESKDRG--LFDFM-G------ | phosphoprotein ECPP44-like | *Gossypium hirsutum* |
| 153 | XP_011088673 | EAKDRGC--FDFM------RK | phosphoprotein ECPP44-like | *Sesamum indicum* |
| 154 | XP_008791981 | EVKDRG--LFDFMG------- | dehydrin COR410-like | *Phoenix dactylifera* |
| 155 | XP_010918213 | EVKDRG--LFDFMG------- | dehydrin COR410-like | *Elaeis guineensis* |
| 156 | XP_010534021 | EVKDRG--LFDFM-GKKEE-- | dehydrin ERD14-like | *Cleome hassleriana* |
| 157 | XP_012465093 | -SKERG--MFDFLG------- | phosphoprotein ECPP44-like | *Gossypium raimondii* |
| 158 | KJB14329 | -SKERG--MFDFLG------- | hypothetical protein B456 002G119600 | *Gossypium raimondii* |
| 159 | XP_017638648 | ESKERG--MFDFL-GKKEDE- | phosphoprotein ECPP44-like | *Gossypium arboreum* |
| 160 | XP_016702923 | ESKERG--MFDFL-GKKEDE- | dehydrin ERD10-like | *Gossypium hirsutum* |
| 161 | XP_010266960 | ETKERG--MFDFFG------- | phosphoprotein ECPP44-like | *Nelumbo nucifera* |
| 162 | XP_016725956 | ESKERG--LFDFMG------- | phosphoprotein ECPP44-like | *Gossypium hirsutum;Gossypium arboreum* |
| 163 | KJB57980 | ESKDRG--LFDFM-GKKEEE- | hypothetical protein B456 009G189500 | *Gossypium raimondii* |
| 164 | XP_012445862 | ESKDRG--LFDFM-GKKEEE- | phosphoprotein ECPP44-like | *Gossypium raimondii* |
| 165 | KJB57981 | ESKDRG--LFDFM-GKKEEE- | hypothetical protein B456 009G189500 | *Gossypium raimondii* |
| 166 | AGC51777 | ESKDRG--LFDFM-GKKEEE- | dehydrin protein | *Manihot esculenta* |
| 167 | ACF15448 | ETTDRG--LFDYFG------- | dehydrin 1 | *Cichorium intybus* |
| 168 | KVI07383 | EKSDRG--LFDYF-GKKDEND | Dehydrin, partial | *Cynara cardunculus var. scolymus* |
| 169 | KNA16389 | ---DRG--LFDCF-GKKEE-- | hypothetical protein SOVF 089520 | *Spinacia oleracea* |
| 170 | ADQ74010 | EATDRG--LFDFI-GKKEEE- | dehydrin, partial | *Solanum ochranthum* |
| 171 | XP_009795068 | EATDRG--LFDFI-GKKEEE- | phosphoprotein ECPP44-like | *Nicotiana sylvestris* |
| 172 | XP_016501573 | EATDRG--LFDFI-GKKEEE- | phosphoprotein ECPP44-like | *Nicotiana tabacum* |
| 173 | AHB20199 | EATDRG--LFDFI-GKKEEE- | dehydrin | *Solanum habrochaites* |
| 174 | NP_001316365 | EATDRG--LFDFI-GKKEEE- | dehydrin | *Solanum lycopersicum* |
| 175 | XP_015074020 | ESTDRG--LFDFI-GKKKEE- | phosphoprotein ECPP44 | *Solanum pennellii* |
| 176 | ADQ73982 | ESTDRG--LFDFI-GKKEEE- | dehydrin, partial | *Solanum peruvianum* |
| 177 | ADQ73999 | ESTDRG--LFDFI-GKKEEE- | dehydrin, partial | *Solanum peruvianum* |
| 178 | ADQ73958 | ESTDRG--LFDFI-GKKEEE- | dehydrin, partial | *Solanum chilense* |
| 179 | ADQ73968 | ESTDRG--LFDFI-GKKEEE- | dehydrin, partial | *Solanum chilense* |
| 180 | ADQ73987 | ESTDRG--LFDFI-GKKEEE- | dehydrin, partial | *Solanum peruvianum* |
| 181 | ADQ73957 | ESTDRG--LFDFI-GKKEEE- | dehydrin, partial | *Solanum chilense* |
| 182 | ADQ73980 | ESTDRG--LFDFI-GKKEEE- | dehydrin, partial | *Solanum peruvianum* |
| 183 | ADQ73984 | ESTDRG--LFDFI-GKKEEE- | dehydrin, partial | *Solanum peruvianum* |
| 184 | ADQ73950 | ESTDRG--LFDFI-GKKEEE- | dehydrin, partial | *Solanum chilense* |
| 185 | ADQ73955 | ESTDRG--LFDFI-GKKEEE- | dehydrin, partial | *Solanum chilense* |
| 186 | ADQ73965 | ESTDRG--LFDFI-GKKEEE- | dehydrin, partial | *Solanum chilense* |
| 187 | ADQ73981 | ESTDRG--LFDFI-GKKEEE- | dehydrin, partial | *Solanum peruvianum* |
| 188 | ADQ73989 | ESTDRG--LFDFI-GKKEEE- | dehydrin, partial | *Solanum peruvianum* |
| 189 | ADQ73953 | ESTDRG--LFDFI-GKKEEE- | dehydrin, partial | *Solanum chilense* |
| 190 | ADQ73986 | ESTDRG--LFDFI-GKKEEE- | dehydrin, partial | *Solanum peruvianum* |
| 191 | ADQ74008 | ESTDRG--LFDFI-GKKEEE- | dehydrin, partial | *Solanum peruvianum* |
| 192 | ADQ73960 | ESTDRG--LFDFI-GKKEEE- | dehydrin, partial | *Solanum chilense* |
| 193 | ADQ73971 | ESTDRG--LFDFI-GKKEEE- | dehydrin, partial | *Solanum chilense* |
| 194 | ADQ73973 | ESTDRG--LFDFI-GKKEEE- | dehydrin, partial | *Solanum chilense* |
| 195 | ADQ73995 | ESTDRG--LFDFI-GKKEEE- | dehydrin, partial | *Solanum peruvianum* |
| 196 | ADQ73997 | ESTDRG--LFDFI-GKKEEE- | dehydrin, partial | *Solanum peruvianum* |
| 197 | ADQ73959 | ESTDRG--LFDFI-GKKEEE- | dehydrin, partial | *Solanum chilense* |
| 198 | ADQ73963 | ESTDRG--LFDFI-GKKEEE- | dehydrin, partial | *Solanum peruvianum* |
| 199 | ADQ73970 | ESTDRG--LFDFI-GKKEEE- | dehydrin, partial | *Solanum chilense* |
| 200 | ADQ74000 | ESTDRG--LFDFI-GKKEEE- | dehydrin, partial | *Solanum peruvianum* |
| 201 | ADQ74001 | ESTDRG--LFDFI-GKKEEE- | dehydrin, partial | *Solanum peruvianum* |
| 202 | ADQ73992 | ESTDRG--LFDFI-GKKEEE- | dehydrin, partial | *Solanum peruvianum* |
| 203 | ADQ73951 | ESTDRG--LFDFI-GKKEEE- | dehydrin, partial | *Solanum chilense* |
| 204 | ADQ73993 | ESTDRG--LFDFI-GKKEEE- | dehydrin, partial | *Solanum peruvianum* |
| 205 | ADQ73990 | ESTDRG--LFDFI-GKKEEE- | dehydrin, partial | *Solanum peruvianum* |
| 206 | ADQ73996 | ESTDRG--LFDFI-GKKEEE- | dehydrin, partial | *Solanum peruvianum* |
| 207 | ADQ73964 | ESTDRG--LFDFI-GKKEEE- | dehydrin, partial | *Solanum chilense* |
| 208 | ADQ73994 | ESTDRG--LFDFI-GKKEEE- | dehydrin, partial | *Solanum peruvianum* |
